# Effectiveness of Some Novel Ionic Liquids on Mild Steel Corrosion Protection in Acidic Environment: Experimental and Theoretical Inspections

**DOI:** 10.3390/ma15062326

**Published:** 2022-03-21

**Authors:** Osama Al-Rashed, Ahmed Abdel Nazeer

**Affiliations:** 1 Chemical Engineering Department, College of Engineering and Petroleum, Kuwait University, Kuwait City 13060, Kuwait; 2 Petroleum Refining & Petrochemicals Research Center, College of Engineering and Petroleum, Kuwait University, Kuwait City 13060, Kuwait; anazeer_nrc@yahoo.com

**Keywords:** mild steel corrosion, ionic liquids, PDP/EIS, SEM/AFM, DFT

## Abstract

Three ionic liquids (ILs)—1-butyl-1-methyl-pyrrolidinium Imidazolate (**BMPyrIM**), 1-butyl-3-methyl-imidazolium Imidazolate (**BMImIM**), and bis(1-butyl-3-methyl-imidazolium Imidazolate) (**BBMImIM**)—were synthesized and examined experimentally and theoretically as potential inhibitors for mild steel corrosion in HCl (1.0 M) solution. To our knowledge, two of the ILs successfully synthesized in our laboratory named **BMPyrIM** and **BBMImIM** are novel. Different electrochemical (potentiodynamic polarization (PDP), electrochemical impedance spectroscopy (EIS)), surface and structural (scanning electron microscopy (SEM), Energy-dispersive X-ray spectroscopy (EDS), Atomic force microscopy (AFM) and Fourier Transform Infrared Spectroscopy (FTIR)) and theoretical (Density functional theory (DFT)) techniques were utilized to confirm their use as efficient environmentally safe inhibitors. These ionic liquids were designed to study the cation effect (imidazolium and pyrrolidinium) and the dimeric effect of the imidazolium-based IL. A pronounced inhibiting effect was recorded using the optimum concentration (5 × 10^−3^ M) of **BBMImIM** with protection efficiency of 98.6% compared to 94.3% and 92.4% for **BMImIM** and **BMPyrIM**, respectively. The investigated ILs act as a mixed-type corrosion inhibitors and their protection obeys Langmuir adsorption isotherm. The results obtained by SEM, EDS and AFM confirmed the mild steel protection by the formation of protective film of the ILs on the steel surface resulted in less damaged surfaces compared with the blank solution. Furthermore, quantum chemical calculations illustrated the electronic structure of the investigated ILs and their optimized adsorptiοn configurations on mild steel surface. The findings from the different techniques helped to provide a supported interpretation of the inhibition mechanism.

## 1. Introduction

Corrosion plays a tremendous and significant role in various industries and, therefore, it causes huge economic losses worldwide [1]. Steel corrosion is commonly acknowledged to have a harmful effect on the structure durability and the mechanical operations. Acidic solutions such as hydrochloric acid (HCl) are extensively used for mild-steel pickling, cleaning, and descaling. Corrosion of mild steel is one of the most significant issues in industry. Corrosion inhibitors can effectively reduce corrosion which suppresses the high rate of metal dissolution [2,3]. These inhibitors form a protective film on metal surfaces to shield the metal from the corrosive solutions and, thus, inhibit corrosion [4]. A good inhibitor should have a great affinity to be adsorbed on the metal surfaces and hence inhibit it through strong binding with the metallic *d*-orbitals [5,6]. It is well known that π electrons; aromatic rings; heteroatoms, such as N, O, and S; and polar functional units such as -OH, -NH_2_, and -SH could significantly improve corrosion inhibition efficiency [7,8]. One of the easiest, most effective and economical protocols is the use of organic inhibitors; however, it is important to design new environmentally friendly corrosion inhibitors [9,10]. For this reason, the green chemistry principles must be followed to reduce the production and the use of toxic chemicals in manufactures [11]. Several attempts were used to develop green corrosion inhibitors. Among them, multicomponent reactions associated with the microwave irradiation [12], chemical drugs, and ionic liquids [13,14]. As many of organic compounds seem to be non-biodegradable and extremely toxic to both humans and the environment [15], ionic liquids were considered as a potential solution to protect metals from corrosion as it meets the criteria of green and sustainable demands.

Ionic liquids are characterized with different unique properties. ILs have excellent electrical conductivity, low volatility and flammability, good chemical and thermal stability, and wide electrochemical window. This made them of great interest in science and engineering fields [16]. ILs are often addressed as green or environmentally friendly because most of them have a negligible vapor pressure, are non-flammable and cannot be inhaled. These features made them safer and more environmentally benign. Some of ILs are not intrinsically green, but they can be made green, and their use in specific processes may lead to improvements that comply with the principles of green chemistry and engineering to use less hazardous chemicals and minimize the risk to human health and the environment. Several books and reviews report the synthesis and applications of ILs as green and environmentally friendly materials [17,18,19]. ILs properties can be modified by modifying their cations or anions [16]. ILs consist of cationic moieties act as electron acceptors and anionic moieties to aid the metallic adsorption of ILs. In general, the anionic moiety of ILs with higher nucleophilic behavior can protect metallic surfaces against corrosion [20].

According to the literature survey, various ILs have been used effectively for inhibiting the mild steel corrosion in different electrolytic media [16,21,22,23]. Recently, we investigated the inhibition effect of three ILs, MPIMI, BMIMI, and HMIMI, for mild-steel dissolution in HCl [24]. We found that the protection efficiency pronouncedly increases with increasing the alkyl chain length. Likhanova et al. studied the cationic effect (imidazolium and pyridinium) on the corrosion inhibition of mild steel in acidic media [25]. Their results showed that the usage of the ionic liquid with imidazolium as a cation instead of pyridinium revealed better inhibition efficiency. Various studies on the application of imidazolium based ILs for the mild-steel protections against corrosion in the acidic environments were previously reported [26,27,28].

There are no reports describing the preparation of ILs with imidazolyl group as an anion to be used in the metallic protection. As far as we know, this will be the first report that describes the corrosion inhibition behavior of this type of ILs. The purpose of this study is to investigate the effect of imidazolate as anion, the role of cationic moiety (imidazolium and pyrrolydinium) and the dimerization of IL monomer on the inhibition behavior of mild steel corrosion in an acidic environment. The considered ionic liquids are 1-butyl-1-methyl-pyrrolidinium Imidazolate (**BMPyrIM**), 1-butyl-3-methyl-imidazolium Imidazolate (**BMImIM**), and bis(1-butyl-3-methyl-imidazolium Imidazolate) (**BBMImIM**). To our knowledge, **BMPyrIM** and **BBMImIM** are new analogies, and they are synthesized for the first time. The inhibition efficiency of the ILs were investigated using potentiodynamic polarization (PDP) and electrochemical impedance spectroscopy (EIS) methods complemented with atomic force microscopy (AFM), scanning electron microscopy (SEM) and the Fourier transform infrared spectroscopy (FTIR) techniques. Quantum chemical studies were also conducted to support the experimental observations as well as to verify the active centers for interactions among the adsorbed inhibitors and the mild steel surface.

## 2. Experimental

### 2.1. Materials and Methods

The working electrode (mild steel) composition is C (0.15%), Mn (0.56%), S (0.36%), P (0.22), V (0.05), Ni (0.04) and the rest Fe. The tested samples were grounded using abrasive paper with grades (400–2000 grit), degreased in acetone, rinsed using bi-distilled water, and air dried. The experiments were carried out in 1.0 M HCl using (HCl 32%) bought from MERCK CHEMICALS and diluted using bi-distilled water.

### 2.2. BBMImIM, BMImIM and BMPyrIM Ionic Liquids

The investigated ionic liquids (**BBMImIM**, **BMImIM** and **BMPyrIM**) were prepared according to a previously described and established procedure [29,30]. For the synthesis of **BMImIM** and **BMPyrIM**, 4.12 moles of 1-methylimidazole or 1-methyl-pyrrolidine were dissolved in 295 mL of dry acetonitrile in a two-necked round-bottomed dry flask. To this flask, 1-bromobutane (4.13 moles) was dropped wisely with stirring for 1 h under argon at 0 °C. The reaction mixtures were then kept at room temperature for further 30 min, followed by raising the temperature to 70 °C for 24 h. After cooling, the reaction mixture was concentrated under vacuum till dryness followed by washing with dry cold acetone and ethyl acetate and concentration in vacuo till dryness until **BMImBr** or **BMPyrBr** was obtained. A methanol solution of NaOH was placed in a flask and stirred followed by adding an equimolar amount of imidazole at room temperature. After 1 hr, an equal mole of **BMImBr** or **BMPyrBr** was added to the mixture. Then, an appropriate amount of anhydrous ether was added three hours later with vigorous stirring at room temperature for 48 h. The reaction mixture was then filtered, washed with anhydrous ether and the filtrates were concentrated in vacuo until dry to yield the desired **BMImIM** and **BMPyrIM** ionic liquids. Mover, for synthesis of **BBMImIM,** 4.12 moles of 1-methylimidazole and excess 1,4-dibromobutane (4.2 moles) were stirred with 200 mL acetonitrile in round 500 mL round-flask at 0 °C for 24 h. The excess of solvent was then removed using a vacuum, and the obtained solid was then washed three times by acetone, followed by vacuum-dried to yield bis(1-butyl-3-methyl-imidazolium bromide) salt (**BBMImBr**). Then, 1.2 moles of **BBMImBr** in a 250 mL single-neck flask was dissolved in 50 mL methanol. To this solution, 2.4 moles of imidazole was added dropwise with stirring at room temperature for 48 h. The reaction mixture was then filtered, washed with anhydrous ether and the filtrates were concentrated in vacuo until dry to afford the desired **BBMImIM** IL.

The name, molecular formula, and molar mass of the examined ILs are included in Table 1.

### 2.3. Electrochemical Measurements

The electrochemical cell is made up of three electrodes: 1 cm^2^ Mild-steel, saturated calomel electrode (SCE), and 1 cm^2^ platinum foil, which serve as working, reference, and counter electrodes, respectively. PDP plots were automatically recorded at a scan rate (1 mVs^−1^) in the potential range of (−0.25 to 0.25 V_SCE_) of the open circuit potential (OCP) and the Stern–Geary method [31] was used to calculate the corrosion current density (i_corr_). EIS tests were carried out in a three-electrode cell at 25 °C in the frequency range (100 kHz to 0.1 Hz) and peak to peak amplitude of 10 mV using alternating current (AC) signals and the spectra were documented after 20 min of immersing in the solution under study. All the electrochemical measurements were performed by 2273 PARSTAT electrochemical workstation Potentiostat (PowerSuite, Ametek Scientific Instrument, Elancourt, France).

### 2.4. Surface Examination

The surface shape of the studied samples was explored after soaking in HCl (1.0 M) solution for 24 h in the absence and existence of 5 × 10^−3^ M of ILs using AFM (VEECO-Nanoscope IV multiple AFM/SPM, Veeco, Santa Barbara, CA, USA) and SEM (JSM-6300 JEOL, Tokyo, Japan), and ATR-FTIR tests with (2000 Perkin-Elmer, Waltham, MA, USA; accomplished at 400–4000 cm^−1^).

### 2.5. Quantum Chemical Calculation

Quantum chemical calculations were achieved using Materials Studio Modeling 7.0 software Accelrys Co. The ionic liquids structures were fully adjusted by DMol^3^ module (version 2016) permitting the solvation effect management via COSMO controls. We used an ab initio quantum approach using double numeric plus polarization (DNP) and becke one parameter (BOP) basis set.

## 3. Results and Discussion

### 3.1. Potentiodynamic Polarization Measurements (PDP)

**Figure 1:**
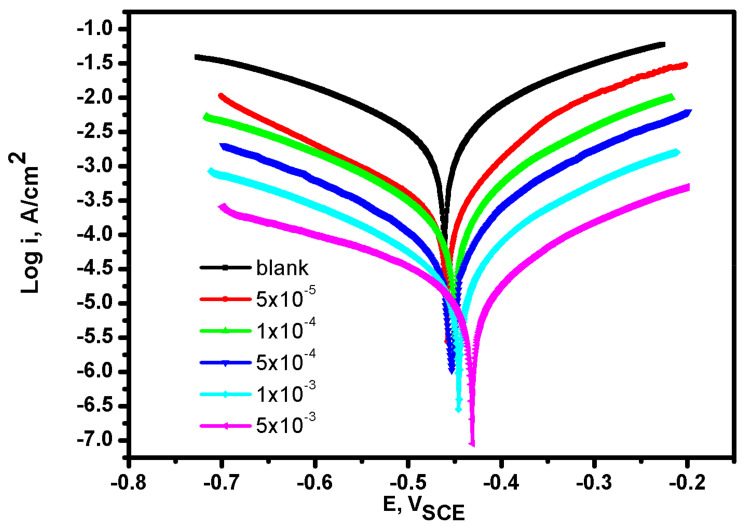
Potentiodynamic polarization curves for mild steel in presence of 1M HCl alone (blank) and containing different concentrations of **BBMImIM** ionic liquid at 25 °C.

The PDP plots of Mild-steel alloy in aerated 1.0 M HCl solution in the absence and with different concentrations of **BBMImIM** are shown in Figure 1. Similar curves in the presence of **BMImIM** and **BMPyrIM** were also recorded but not shown. The measurements were carried out after 20 min to reach a steady state potential at a scan rate of 1.00 mVs^−1^. Results of corrosion potential (E_corr_) and i_corr_ obtained by extrapolating the cathodic (β_c_) and anodic (β_a_) Tafel slopes are presented in Table 2. The obtained results demonstrate that in the existence of 1M HCl solution free from ILs, the corrosion current density is relatively high in the cathodic part due to hydrogen evolution reaction. In case of using **BBMImIM** as an inhibitor, the anodic and cathodic polarization plots were markedly altered to more noble potentials compared to bare solution. The shift in corrosion potential is also increased by increasing the **BBMImIM** concentration. Moreover, the i_corr_ is decreased by raising the IL concentration as presented in Table 2.

The inhibition efficacy of the examined ILs to control the mild steel dissolution was estimated from the polarization curves by the following equation [32]:

η_pp_ % = θ × 100 = [(j_corr_ − j′_corr_)/j_corr_] × 100 (1)

where j_corr_ and j′_corr_ are the corrosion current density (µA cm^−2^) with and without the examined ILs, respectively.

Using Equation (1) and according to the data in Table 2, the inhibition efficacy raises upon increasing the IL concentration. The greatest inhibition efficacies obtained for each of the studied inhibitors were 98.62%, 94.33% and 92.32% in presence of 5 × 10^−3^ M of **BBMImIM**, **BMImIM** and **BMPyrIM**, respectively. The results confirmed that the investigated ILs have an impact on both anodic steel dissolving and cathodic hydrogen-evolution processes which confirm that the explored ILs are effective inhibitors for steel dissolution in the acidic solutions.

### 3.2. Adsorption Isotherm

To define the adsorption type of the synthesized ionic liquids on the mild steel interface, several isotherms (Frumkin, Freundlich, Langmuir, and Temkin) were used. In our work, Langmuir isotherm has exhibited an outstanding match based on regression coefficient values that were near unity. The θ results estimated from the PDP methodology were evaluated using the Langmuir isotherm [33], which could be expressed as:

C/θ = 1/K_ads_ + C (2)

where C denotes the IL concentration while K_ads_ is the constant of adsorption–desorption.

**Figure 2:**
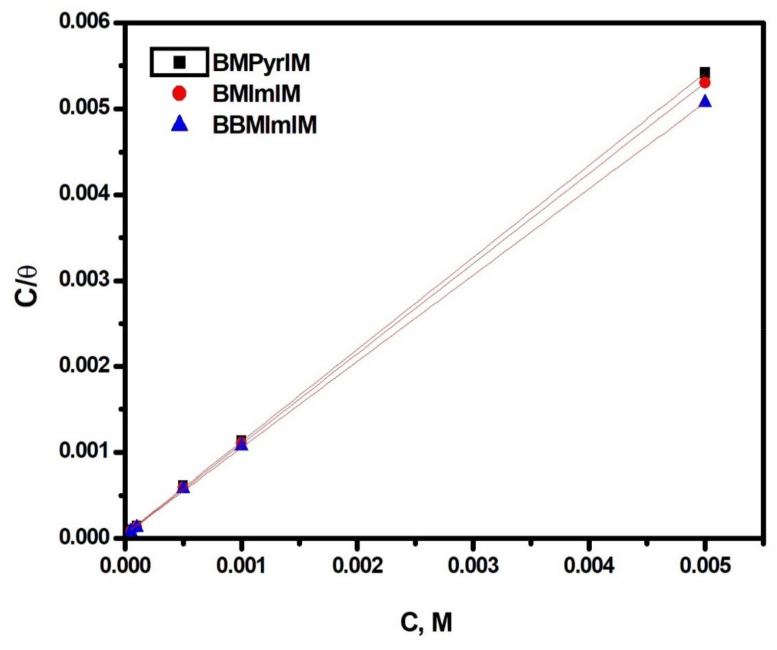
Langmuir isotherm for the studied ionic liquids (**BMPyrIM**, **BMImIM** and **BBMImIM**) adsorption on mild steel in 1 M HCl at 25 °C.

Figure 2 displays a linear plot of C/θ vs. C according to the Langmuir-isotherm with a regression constant (R^2^) and slope close to unity (Table 3). The intercept of the plot was used to obtain the K_ads_ values for the examined ILs, whereas the standard free energy (G_ads_) values were produced as follows [34]:

K_ads_ = 1/55.5 exp [−ΔG°_ads_/RT] (3)

where 55.5 denotes H_2_O concentration in solution, T is the absolute temperature and R is the gas constant.

The greater values of K_ads_ given in Table 3 for the examined ILs manifest the significant interaction of the ILs molecules with the mild steel interface. Compared to the other ILs, **BBMImIM** showed the highest K_ads_ value that confirms its best inhibition effectiveness. Furthermore, the negative ΔG_ads_ values presented in Table 3 reveal the spontaneous development of the studied ILs on the Mild steel interface [35]. All the estimated ΔG_ads_ values were between −20 kJ mol^−1^ and −40 kJ mol^−1^ indicating that the interaction among the ILs and the steel interface included both physical and chemical adsorption. The physical adsorption depends on the electrostatic interaction among the charged surface of mild steel with the charged molecules of ILs [36]. While the chemical adsorption was accompanied to the removal of H_2_O molecules from the metallic interface [37].

### 3.3. EIS Study

**Figure 3:**
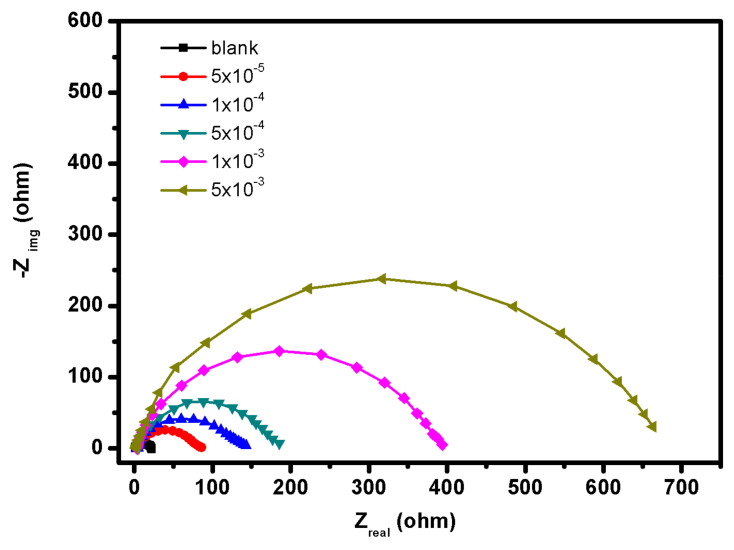
Nyquist plots for mild steel in presence of 1.0 M HCl alone and containing different concentrations of **BBMImIM** ionic liquid at 25 °C.

EIS test was utilized to validate the electrochemical data gathered using the PDP approach. Figure 3 represents the Nyquist curves of mild steel corrosion in 1.0 M HCl medium without and in the existence of diverse concentrations of **BBMImIM** (comparable plots for **BMImIM** and **BMPyrIM** are not shown). It is obvious from these figures that the Nyquist plots depict depressed semicircle which may attributed to the roughness produced from the alloy surface and the adsorption of ILs resulted in the formation of porous layers [38]. When Nyquist plots were analyzed electrochemically, the difference among real impedance at higher and lower frequencies generates charge transfer. Furthermore, following the addition of the ILs, the diameters of the semicircles of Nyquist plots showed a significant increase than in the absence of the ILs, and the diameters were steadily enlarged by raising the **BBMImIM** concentration.

These findings show that the dissolution rate of mild steel surfaces in the existence of ILs is pronouncedly reduced when the corrosive action of acidic solution is controlled. This is owing to the formation of a protecting layer on the steel interface which effectively suppressed its corrosion [39].

**Figure 4:**
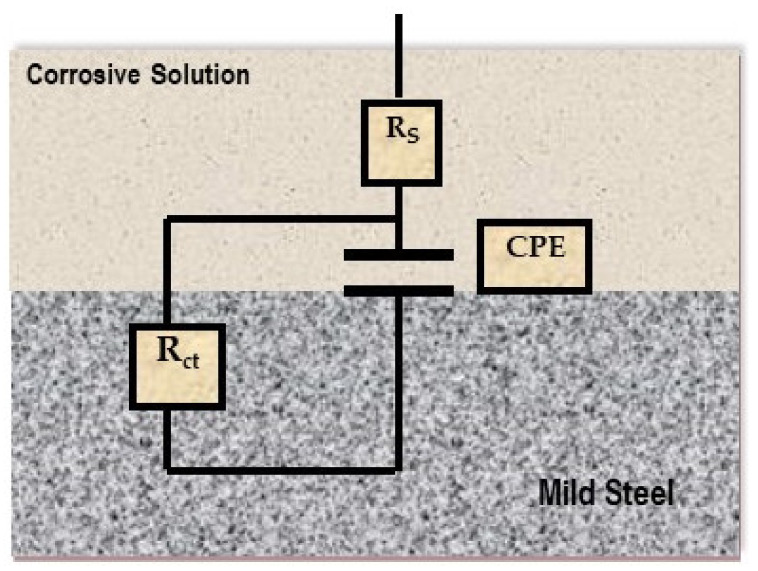
Equivalent circuit used for fitting the data.

The designed circuit for fitting the EIS results is shown in Figure 4, that was successfully utilized to assess EIS data for mild steel in HCl solution [40]. The R_s_, R_ct_ and CPE represent the solution resistance, charge transfer resistance and the double layer capacitance, respectively. It is essential to use CPE as a replacement for capacitance due to metal/inhibitor interactions and considering inhomogeneity and roughness. The CPE impedance was obtained as follows [41]:

Z(CPE) = 1/[Y^0^(*j* ω)^n^] (4)

where Y^0^ is the magnitude of CPE and n signifies its exponent, *j* is the imaginary number and ω is the angular frequency.

The values of n could be used to determine the topology of the deteriorated metal surfaces in corrosive medium, with a larger value indicating better surface smoothness. The value of n ranges from 0.0 to 1.0. When this number near unity, it indicates that the capacitor is in an ideal condition and the metal interface is highly homogenous. If n value is 0, the CPE signifies a resistive behavior but, as n = 1/2 and Y^0^ = W, this indicates Warburg impedance.

The inhibition efficiency (η_EIS_) for the corrosion of mild steel in HCl can be obtained from the R_ct_ values as follow [42]:

η_EIS_ % = [(R_ct(inh)_ − R_ct_)/R_ct(inh)_] × 100 (5)

where R_ct_ and R_ct(inh)_ represent the charge-transfer resistances in the uninhibited and the inhibited solutions, respectively. The recorded EIS data are displayed in Table 4.

It is evident that the charge transfer resistance values have clearly improved as the **BBMImIM** concentration is raised, whereas the C_dl_ values decreased. This shows that the adsorbed IL molecules are gradually increased on the mild steel surface, resulting in a decrease in the active sites necessary for the dissolving reaction. The rise in R_ct_ indicates a significant limitation of charge transfer through the protective layer with the adsorbed ILs on the metallic surface, reflecting the high resistance to acidic attack and, as a result, inhibiting the dissolution process. Moreover, the C_dl_ values were diminished owing to a drop in the dielectric constant or the improvement in the electrical double layer thickness attributed to the ILs adsorption on the steel surface [43].

**Figure 5:**
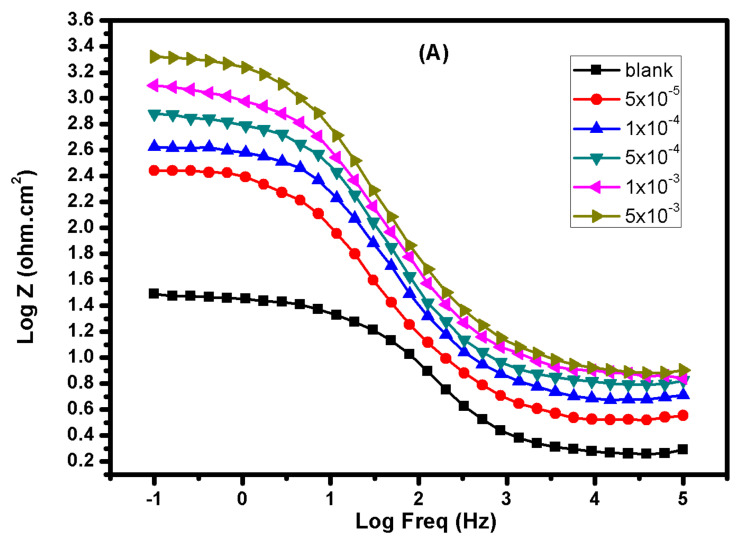

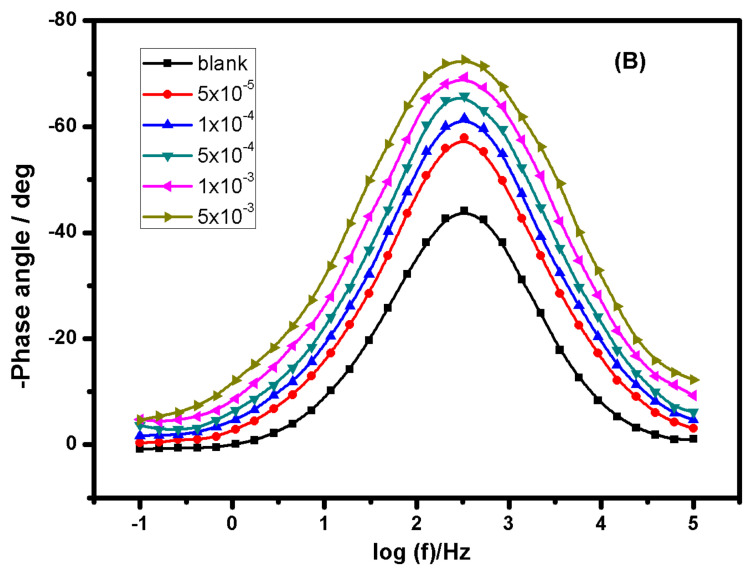
Bode (**A**) and phase angle (**B**) plots for mild steel in presence of 1.0 M HCl alone and containing different concentrations of **BBMImIM** ionic liquid at 25 °C.

The Bode plots for mild steel in 1.0 M HCl solution with and without various concentrations of **BBMImIM** are presented in Figure 5. From this figure, it is obvious that the existence of the studied ILs resulted in raising the phase angle in comparison with the blank solution. The higher value of the phase angle in the presence of **BBMImIM** confirmed the low roughness of the steel-surface as well as the effective adsorption of the IL on the surface with protective layer growth. In addition, the presence of **BBMImIM** showed higher resistance at the low frequency region and it was increasing with the rise of **BBMImIM** concentration. In summary, the findings recorded from PDP method agreed with the EIS results, approving the validity of both approaches for assessing the tested ILs.

### 3.4. Surface Characterization of Mild Steel

#### 3.4.1. Scanning Electron Microscopy Study

**Figure 6:**
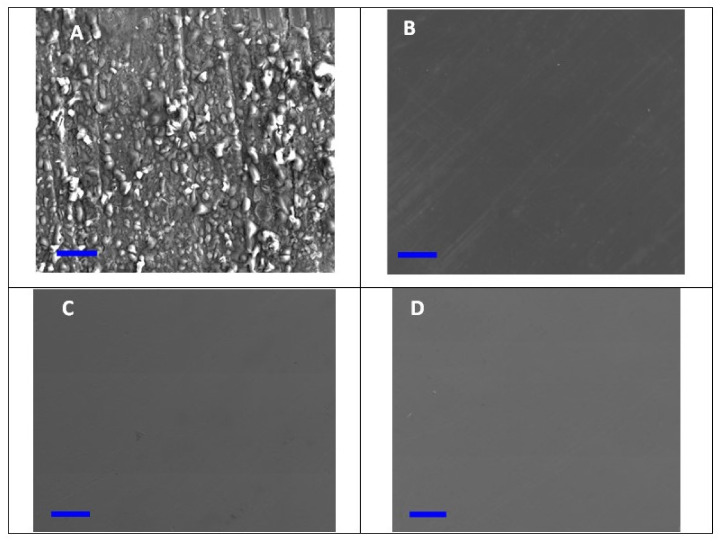
Scanning electron microscopy (SEM) images of the mild-steel in 1.0 M HCl alone (**A**) and containing 5 × 10^−3^ M of **BMPyrIM** (**B**), **BMImIM** (**C**) and **BBMImIM** (**D**) ionic liquids at 25 °C for 24 h. Scale bar is 10 µm.

Figure 6A–D display the SEM pictures of the mild steel surface after soaking in 1.0 M HCl solution for one day in the absence and existence of 5 × 10^−3^ M of the investigated ILs (**BMPyrIM**, **BMImIM** and **BBMImIM**). The surface morphology of the mild steel exposed to the 1.0 M HCl solution alone (Figure 6A) was degraded, rough and porous with the evidence of dense film of corrosion products due to the steel degradation in the corrosive medium. In contrast, the addition of the examined **BMPyrIM**, **BMImIM** and **BBMImIM** ILs in the acidic media, considerably inhibited the mild steel surface and resulted in a smoother surface as shown in Figure 6B–D). The steel surface was smoother in the existence of **BBMImIM** compared to **BMImIM** and **BMPyrIM**, respectively. Consequently, **BBMImIM**, **BMPyrIM** and **BMImIM** can obviously suppress the aggressive attack of the HCl solution along with the development of a protective layer on the mild steel surface that resulted in a pronouncedly high inhibition from dissolution.

#### 3.4.2. Energy-Dispersive X-ray Spectroscopy

**Figure 7:**
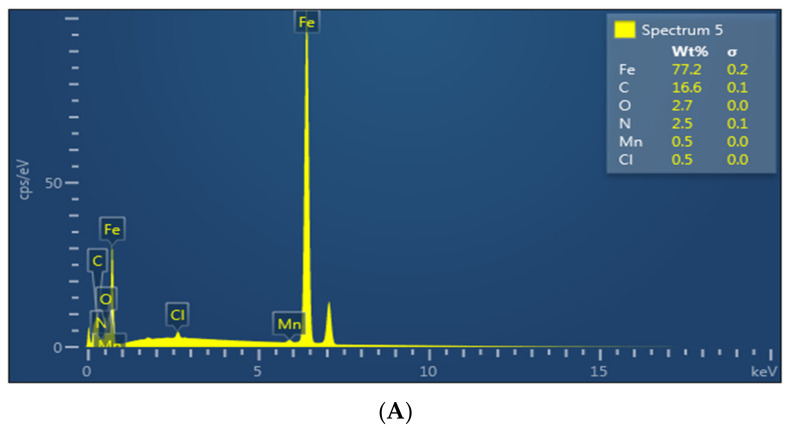

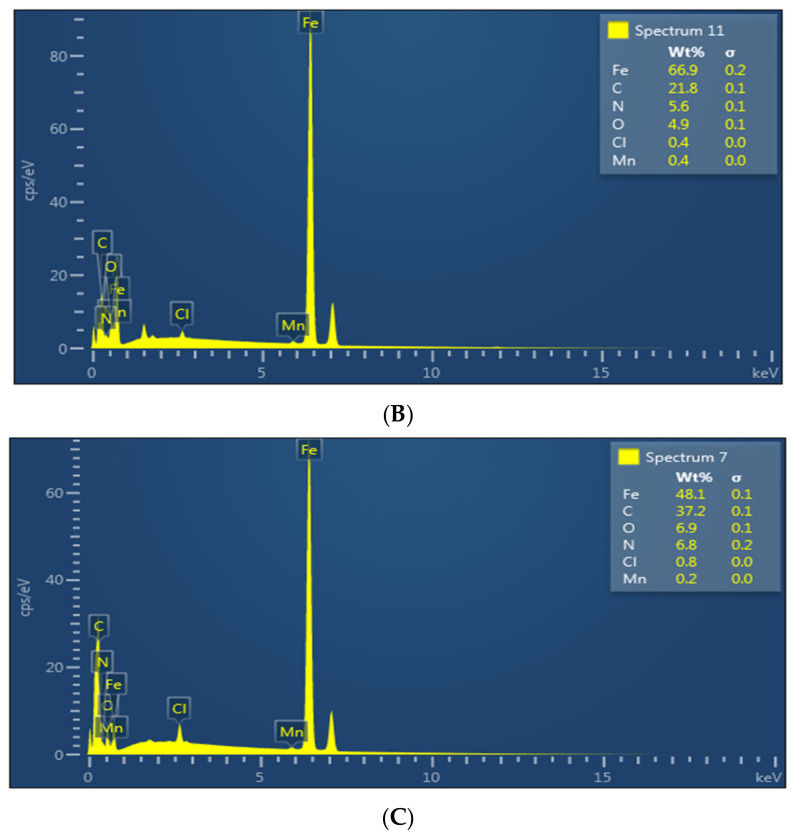
Energy-dispersive X-ray spectroscopy (EDS) images of the mild-steel in 1.0 M HCl containing 5 × 10^−3^ M of **BMPyrIM** (**A**), **BMImIM** (**B**) and **BBMImIM** (**C**) ionic liquids at 25 °C after immersion for 24 h.

Figure 7 displays the EDS analyses of mild steel surface after dipping it in 1.0 M HCl medium containing the optimum concentration (5 × 10^−3^ M) of the investigated ionic liquids for 24 h at room temperature. The spectra contain Fe which is the main element present in the mild steel; additionally, it contains Cl from HCl solution and oxygen from the oxides formed. The presence of C and N peaks in the EDS spectra confirms the adsorption of the ILs on the mild steel surface. The intensity of the signal height of C and N is higher in the presence of **BBMImIM** than in case of **BMImIM** and finally **BMPyrIM** which proves the formation of more intense film in presence of **BBMImIM** that is in harmony with the SEM observations.

#### 3.4.3. Atomic Force Microscopy Analysis

**Figure 8:**
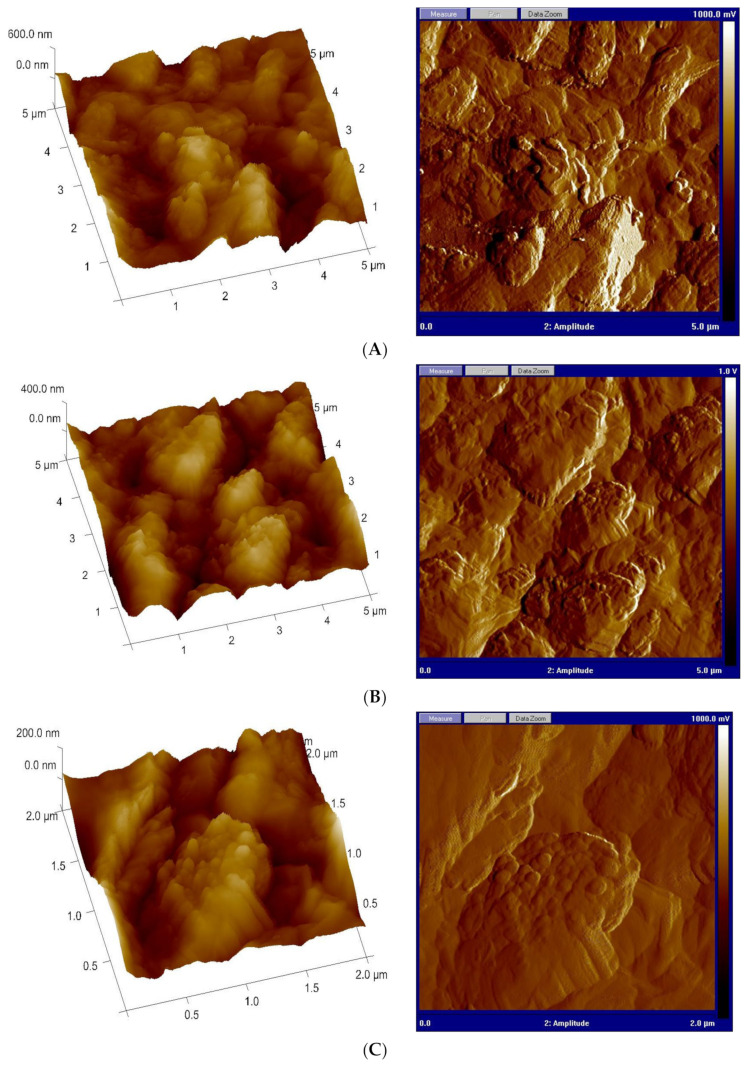

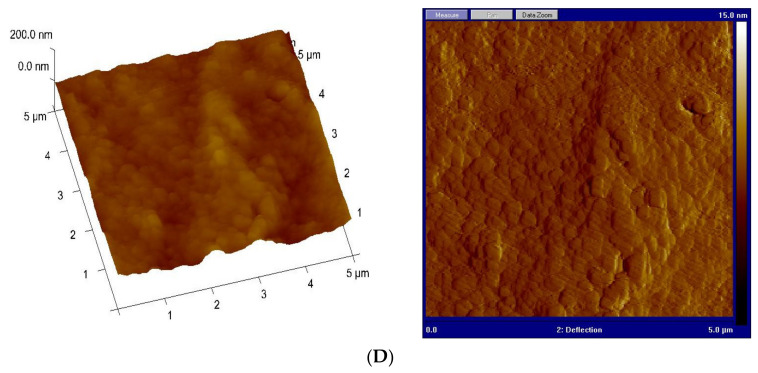
The 2d and 3d Atomic force microscopy (AFM) micrographs of mild steel surface in 1.0 M HCl solution in the absence (**A**) and presence of 5 × 10^−3^ M of **BMPyrIM** (**B**), **BMImIM** (**C**) and **BBMImIM** (**D**) after 24 h immersion.

AFM analysis is regarded as a crucial characterization technology for investigating the morphology of the metallic surface and evaluating the dissolution process that happens at the metal/electrolyte contact. Figure 8A–D display the 2d and 3d AFM images of mild steel dipped in 1.0 M HCl for 24 h in the absence and existence of the optimum concentration (5 × 10^−3^ M) of the investigated ILs (**BMPyrIM**, **BMImIM** and **BBMImIM**). Apparently, the AFM images collected in the absence of the investigated ILs revealed a damaged and rough surface with various pits caused by the aggressive ions action (Figure 8A). In addition, reasonably smoother surfaces were achieved when the studied **BMPyrIM**, **BMImIM** and **BBMImIM** ILs were used, as displayed in Figure 8B–D, respectively. These observations obviously reveal that mild steel degradation might be controlled and reduced. Furthermore, the addition of the examined ILs showed the creation of protective thin layer on the steel surface with the following inhibition trend: **BBMImIM** > **BMImIM** > **BMPyrIM**.

#### 3.4.4. ATR-FTIR Study

**Figure 9:**
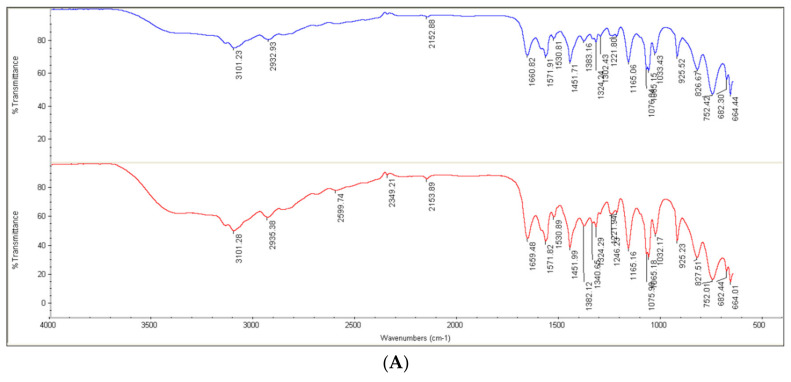

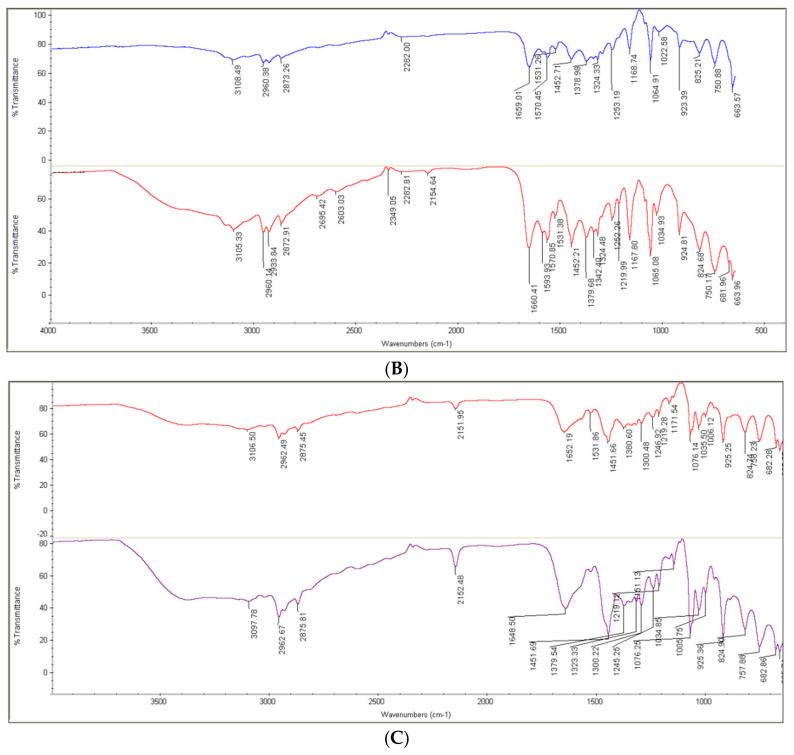
Fourier Transform Infrared Spectroscopy (ATR-FTIR) spectrum for **BBMImIM** (**A**), **BMImIM** (**B**) and **BMPyrIM** (**C**) as a pure and adsorbed on the mild steel surface after immersion for 24 h.

The ATR-FTIR spectra of pure **BBMImIM**, **BMImIM** and **BMPyrIM** ionic liquids and the adsorbed ILs on mild steel surface in the range from 4000 to 400 cm^−1^ are presented in Figure 9. The peak around 3600–3200 cm^−1^ is assigned to the vibrational −OH from water, whereas the peaks between 3200 and 3100 cm^−1^ are assigned to the C–H stretching vibrations of the imidazole and pyrrolydinium rings [44]. The bands obtained among 3000 and 2800 cm^−1^ are matched to the C–H stretching vibrations of the alkyl groups attached to the imidazolium and pyrrolydinium rings. The peaks at 1650–1500 cm^−1^ and 1451 cm^−1^ are characteristics to the skeletal vibration of the rings [45], while the peak at about 1253 cm^−1^ assigned to symmetric C–H vibration of imidazolium and pyrrolydinium rings. Furthermore, the peaks at about 1168 and 827 cm^−1^ are corresponded to in-plane C-H bending vibration peaks of imidazolium ring whereas the peak at 623 cm^−1^ is the characteristic of the CH_2_ plane rocking vibration of the alkyl chain.

From the analysis of the FTIR spectra, it is clear that there are nearly similar peaks in the same region of spectrums in case of the adsorbed layer on the mild steel surface compared with the pure IR spectrum of the corresponding IL. There is a little shift and decease in the intensity of the bands owing to the chemical interaction among the ILs and the steel surface. These findings suggest the adsorption of the examined ILs on the steel surface confirming their pronounced protection efficiency.

### 3.5. Quantum Chemical Study

As per Frontier molecular orbital theory (FMO), the highest occupied molecular orbital (HOMO) and lowest unoccupied molecular orbital (LUMO) can be readily associated to the inhibitor’s protective efficacy [46]. Therefore, using inhibitors with high E_HOMO_ and low E_LUMO_ values enhances the corrosion protection performance. Because E_HOMO_ is connected to the inhibitor’s potential for donating electrons, raising E_HOMO_ values makes it simpler for electron donation to the metal’s vacant d-orbital. While E_LUMO_ is related to a molecule’s capacity to receive electrons. Consequently, the smaller E_LUMO_ value, the higher probability a molecule is to receive electrons. As a result, an inhibitor’s inhibition efficacy typically rises as E_HOMO_ values increase and E_LUMO_ values diminish. Additionally, a reduced energy difference among LUMO and HOMO energy (∆E) for an ionic liquid suggests its high deterioration protection efficacy.

**Figure 10:**
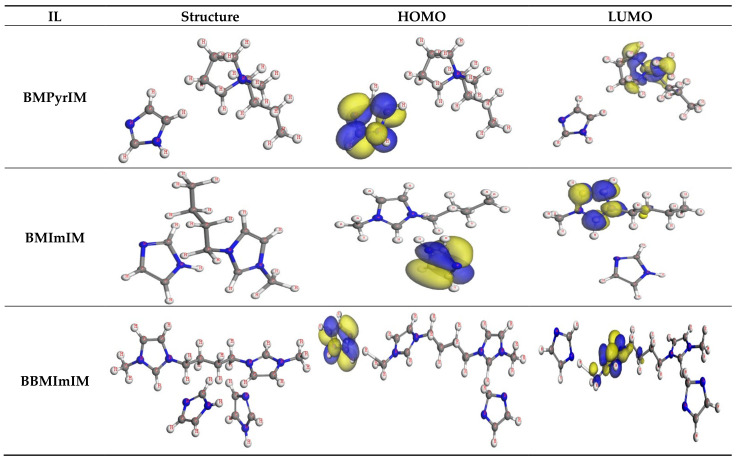
The optimized molecular structures, HOMO and LUMO of the investigated ILs (**BMPyrIM**, **BMImIM** and **BBMImIM**). Blue and yellow isosurfaces of the HOMO and LUMO denote positive and negative wavefunction phases, respectively, using Dmol^3^ module.

The **BBMImIM** ionic liquid showed the largest value of E_HOMO_ in comparison with the **BMImIM** and **BMPyrIM** as revealed in Table 5. As presented in Figure 10, the HOMO level for the **BBMImIM** IL was clearly positioned along the imidazolyl ring, indicating that the nitrogen atoms are the desired place for electrophilic attacks on the steel interface. This will endorse the **BBMImIM** potential to be adsorbed on the metallic surface and boost the protection effectiveness, which is consistent with the experimental findings. Furthermore, the smaller E_LUMO_ value for **BBMImIM** fits the results well, as shown in Table 5. The energy gap (ΔE) is vital to validate the theoretical capability of the IL structure for the corrosion prevention that rises as the ΔE is decreased [47]. From Table 5, **BBMImIM** showed the lowest ΔE value compared to **BMImIM** and **BMPyrIM**, which confirms that **BBMImIM** is more likely to be adsorbed on the steel interface. The findings also reveal a strong association among both the molecular structure of the examined ILs and their propensity for protecting the steel surface in the aggressive solution. As the molecular weight of the inhibitor increase, the efficacy of inhibition enhances since the contact area between the ILs molecules and the steel surface rises. Therefore, **BBMImIM** has the greatest surface area and, hence, greater protecting effectiveness. Additionally, the obtained dipole moment (μ) values were used to distinguish the ILs under consideration. It is known that IL with a high μ value can facilitate the adsorption action and hence the inhibition process owing to a strong dipole–dipole interaction among the IL and the steel surface [29]. According to the results in Table 5, **BBMImIM** showed the highest μ value when compared to the other ionic liquids, which is consistent with the other obtained results.

### 3.6. Corrosion Inhibition Mechanism

The corrosion inhibition mechanism of mild steel in the HCl solution in the existence of the examined ILs can be explained as follows: 

Generally, in the acidic solutions and the absence of inhibitors, the mild-steel surface has been shown to be positively charged and can easily be corroded owing to the aggressive chloride ions present in the solution. The use of organic inhibitors can suppress metallic dissolution by being adsorbed on the metal surface through the functional groups and the π electrons that exist on these inhibitors. According to the literature, ionic liquids, like most of the corrosion inhibitors, inhibit the metals and alloys corrosion via blocking the cathodic and anodic reaction sites.

In this study, the existence of imidazolium and pyrrolidinium-based ionic liquids resulted in slowing down the anodic dissolution reactions rate by the growth of a protecting layer capable of isolating the metallic surface away from the acidic medium as follows [48]:

Fe(H_2_O)_n_ + 2Cl^−^ → [Fe(H_2_O)_n_ 2Cl^−^] (6)

[Fe(H_2_O)_n_ 2Cl^−^] + [BMIm]^+^ or [BMP]^+^ → [Fe(H_2_O)_n_ 2Cl^−^][BMIm]^+^ or [Fe(H_2_O)_n_ 2Cl^−^][BMPyr]^+^ (7)

The inhibiting effect of the examined ILs resulted from covering the steel surface with the aromatic-ring and the butyl chains to form an efficient layer at the steel/solution boundary covering the steel surface’s active centers. The imidazole’s nitrogen and oxygen atoms have the ability to donate lone-pairs of electrons to the vacant d-orbital of steel producing a coordination bond and also, the imidazole π-electrons are bonded to the steel surface’s vacant d-orbital to facilitate their protection. Furthermore, the possibilities of hydrogen bonding among the imidazolium ring hydrogen and the counter ions in addition to H_2_O molecules assist the inhibition action.

In the absence of ILs, the reactions at the cathodic part were involved in the hydrogen gas evolution as in the following equation:

H^+^ + e^−^ → ½ H_2_ (8)

While in the existence of the investigated ILs, the cationic moiety of the ILs (imidazolium or pyrrolydinium moiety) were adsorbed at the cathodic site and thus competing with the H^+^ ions to suppress the cathodic processes as follows:

Fe + [BMIm]^+^ or [BMPyr]^+^ → (Fe[BMIm])^+^ or (Fe[BMPyr])^+^ (9)

(Fe[BMIm])^+^ or (Fe[BMPyr])^+^ + e^−^ → (Fe[BMIm]) or (Fe[BMPyr]) (10)

Moreover, the imidazolyl anion can act as counter ion among the steel surface and the imidazolium or pyrrolydinium cations, which assist the physical adsorption of the ILs on the steel surface via the electrostatic interactions [25]. Since the inhibiting efficiency depends on both the cathodic and anodic parts of the ionic liquid molecule, **BBMImIM** ionic liquid showed the highest level of protection due to the higher surface coverage owing to its dimeric structure and the higher molar mass which resulted in superior protection.

In order to explain the cation effect, the lower protection efficiency obtained using the pyrrolydinium cation compared with the imidazolium cation can be interpreted by the positive charge localization on the nitrogen atom of the pyrrolydinium cation that led to the repulsion with the positively charged steel surface in the acidic solution resulting in a lower protection in comparison with the imidazolium cation. The experimental and the theoretical results support each other confirming the order of protection of the examined ILs as follows: **BBMImIM** > **BMImIM** > **BMPyrIM**. Finally, according to the fascinating physiochemical properties of ILs, including non-toxicity, high conductivity, non-flammability, less volatility, and high thermal and chemical stability, they can be used instead of traditional volatile (toxic) compounds as corrosion inhibitors in several industries.

## 4. Conclusions

Different experimental, surface analysis and theoretical techniques were applied to investigate the protection efficiency of mild steel dissolution in 1.0 M HCl using ionic liquids namely **BBMImIM**, **BMImIM** and **BMPyrIM**. The results demonstrate that the examined ILs were effectively able to protect the mild steel surface via their physisorption and chemisorption on the steel surface obeying Langmuir adsorption isotherm. The examined ILs are acting as mixed-type inhibitors controlling the anodic and cathodic corrosion mechanisms. The inhibition efficiency of the ILs was increased as the concentration increased up to 5 × 10^−3^ M resulted in the highest protection of 98.6% using **BBMImIM** followed by 94.3% and 92.4% in the presence of **BMImIM** and **BMPyrIM**, respectively. SEM, AFM and ATR-FTIR graphs proved the passive film formation on the steel surface which mitigates the steel corrosion in presence of ILs and these results were supported using the EDS spectra which verified the existence of the carbon and the nitrogen of the ILs on the steel surface. The electrochemical, surface analysis and theoretical results match each other, suggesting that the investigated ILs have excellent corrosion inhibition performance for the mild steel surface in acidic solution.

## Figures and Tables

**Table 1:** The names, chemical structures, and molecular weights of the investigated ionic liquids.

IL Name	Chemical Structure	Molar Mass
bis(1-butyl-3-methyl-imidazolium Imidazolate) **BBMImIM**	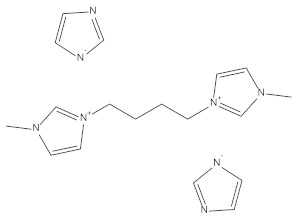	354.45
1-butyl-3-methyl-imidazolium Imidazolate **BMImIM**	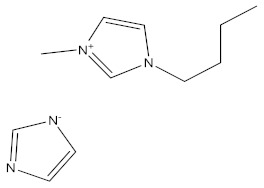	206.29
1-butyl-1-methyl-pyrrolidinium Imidazolate **BMPyrIM**	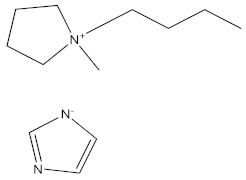	209.33

**Table 2:** Electrochemical parameters obtained from potentiodynamic polarization measurements of mild steel alloy in 1 M HCl in the absence and presence of various concentrations of the investigated ionic liquids at 25 °C.

	Conc., M	j_corr._, µA cm^−2^	−E_corr._, mV	−β_c_, mV dec.^−1^	β_a_, mV dec.^−1^	θ	η_pp_ %
Blank	-	4776	461	283	198	--	--
**BMPyrIM**	5 × 10^−5^	2316	463	196	109	0.5151	51.51
	1 × 10^−4^	1414	465	191	103	0.7039	70.39
	5 × 10^−4^	869	468	163	92	0.8180	81.80
	1 × 10^−3^	558	474	142	78	0.8832	88.32
	5 × 10^−3^	367	477	138	75	0.9232	92.32
**BMImIM**	5 × 10^−5^	2029	459	212	192	0.5752	57.52
	1 × 10^−4^	1284	455	207	188	0.7312	73.12
	5 × 10^−4^	755	454	199	143	0.8419	84.19
	1 × 10^−3^	436	450	187	100	0.9087	90.87
	5 × 10^−3^	271	449	153	77	0.9433	94.33
**BBMImIM**	5 × 10^−5^	1809	458	210	178	0.6212	62.12
	1 × 10^−4^	1085	453	200	154	0.7728	77.28
	5 × 10^−4^	644	450	180	143	0.8652	86.52
	1 × 10^−3^	323	446	174	117	0.9324	93.24
	5 × 10^−3^	66	431	170	79	0.9862	98.62

**Table 3:** Regression constant (R^2^), equilibrium constant (K_ads_), and free energy of adsorption (ΔG_ads_) of the studied ionic liquids for the corrosion of mild steel in 1 M HCl at 25 °C.

Comp.	Langmuir Isotherm		
	R^2^	K_ads_ (M^−1^)	−ΔG_ads_ (kJ mol^−1^)
**BMPyrIM**	0.99975	29,877	34.91
**BMImIM**	0.99983	81,234	37.23
**BBMImIM**	0.99991	98,766	39.98

**Table 4:** Data attained by electrochemical impedance spectroscopy (EIS) technique for the corrosion of mild steel alloy in 1.0 M HCl in the absence and presence of different concentrations of the studied ILs at 25 °C. **χ^2^** is the goodness of the fit.

	Conc., M	R_s_ Ω cm^2^	R_ct_ Ω cm^2^	n	C_dl_ µF cm^−2^	θ	η_EIS_ %	χ^2^
Blank		1.52	23.3	0.833	102.3	--	--	0.00024
**BMPyrIM**	5 × 10^−5^	1.36	45.9	0.926	81.9	0.4924	49.24	0.00049
	1 × 10^−4^	1.32	81.7	0.911	64.7	0.7148	71.48	0.00051
	5 × 10^−4^	1.30	111.6	0.920	57.3	0.7912	79.12	0.00044
	1 × 10^−3^	1.35	182.5	0.914	47.5	0.8723	87.23	0.00039
	5 × 10^−3^	1.26	266.1	0.910	44.6	0.9124	91.24	0.00031
**BMImIM**	5 × 10^−5^	1.44	57.8	0.932	74.4	0.5969	59.69	0.00047
	1 × 10^−4^	1.32	91.7	0.925	61.5	0.7459	74.59	0.00045
	5 × 10^−4^	1.31	151.6	0.915	53.4	0.8463	84.63	0.00039
	1 × 10^−3^	1.38	239.3	0.909	44.3	0.9026	90.26	0.00033
	5 × 10^−3^	1.29	381.1	0.912	37.7	0.9389	93.89	0.00026
**BBMImIM**	5 × 10^−5^	1.42	64.8	0.917	62.2	0.6404	64.04	0.00042
	1 × 10^−4^	1.39	103.1	0.908	53.1	0.7740	77.40	0.00033
	5 × 10^−4^	1.24	165.6	0.902	41.4	0.8593	85.93	0.00030
	1 × 10^−3^	1.29	329.6	0.899	32.2	0.9293	92.93	0.00031
	5 × 10^−3^	1.27	781.3	0.905	29.5	0.9702	97.02	0.00029

**Table 5:** The calculated quantum chemical parameters of the examined ionic liquids.

	E_HOMO_, eV	E_LUMO_, eV	ΔE, eV	μ (Debye)
**BMPyrIM**	−6.09	−1.38	4.71	9.64
**BMImIM**	−5.69	−1.63	4.06	13.22
**BBMImIM**	−5.44	−1.82	3.62	16.89

## Data Availability

Not applicable.
